# Predicting vital wheat gluten quality using the gluten aggregation test and the microscale extension test

**DOI:** 10.1016/j.crfs.2020.11.004

**Published:** 2020-12-03

**Authors:** Marina Schopf, Katharina Anne Scherf

**Affiliations:** a Leibniz-Institute for Food Systems Biology at the Technical University of Munich, Lise-Meitner-Straße 34, 85354, Freising, Germany; b Department of Bioactive and Functional Food Chemistry, Institute of Applied Biosciences, Karlsruhe Institute of Technology (KIT), Adenauerring 20a, 76131, Karlsruhe, Germany

**Keywords:** Baking, Gluten, GlutoPeak test, Microscale extension, Wheat

## Abstract

Vital gluten is a by-product of wheat starch production and commonly used in bread making, but its quality is difficult to predict. The most accurate method to determine vital gluten quality is the baking experiment, but this approach is time- and labor-intensive. Therefore, the aim was to identify faster and easier ways to predict vital gluten quality. Three different approaches, the gliadin/glutenin ratio, the gluten aggregation test and the microscale extension test, were assessed for their predictive value regarding the baking performance of 46 vital gluten samples using two recipes. Hierarchical clustering classified the vital gluten samples into 23 samples with good, 15 with medium and eight with poor quality. Protein-related parameters, such as the gliadin/glutenin ratio, were not reliable to predict gluten quality, because the correlations to the bread volumes were weak. The gluten aggregation test and the microscale extension test were reliable methods to predict vital gluten quality for use in baking based on a scoring system. Both methods need less material, time and labor compared to baking experiments. Especially, maximum torque, peak maximum time, the ratio between peak30 and peak180 as well as the corresponding distance at maximum resistance to extension seem to be suitable alternatives to predict vital gluten quality.

## Highlights

• Vital gluten, a by-product of wheat starch production, is commonly used in foods
• Its functionality is predicted using time- and labor-intensive baking tests
• The gluten aggregation and microscale extension test were quality predictors
• Maximum torque, peak maximum time, and specific peak area ratios were most useful

## Graphical abstract

**Figure:**
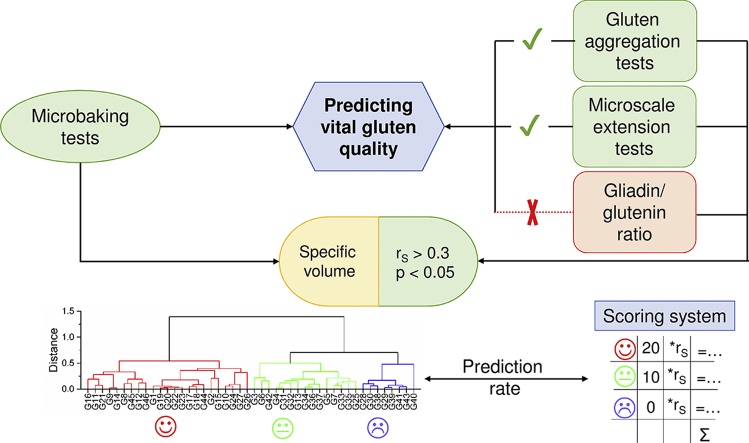

## 1 Introduction

Wheat gluten contains storage proteins, which are a complex mixture of more than one hundred single proteins, composed of alcohol-soluble gliadins (GLIA) and alcohol-insoluble glutenins (GLUT) (Shewry, 2019). Gluten is typically separated from wheat flour by washing out starch and water-soluble components (Bailey, 1941). This simple experiment represents the beginning of gluten extraction and is still the basis of modern day processes like the Martin process, the batter process or variations thereof (Van der Borght, Goesaert, Veraverbeke and Delcour, 2005). After separating wet gluten from wheat starch, a drying step at low temperatures is necessary to facilitate handling, ensure microbiological stability and retain functional properties like cohesivity, elasticity, viscosity, and extensibility (Weegels et al., 1994). The resulting dry powder is called vital gluten and is defined in the CODEX STAN 163-1987, 2001 as a wheat protein product that contains at least 80% crude protein (N ​× ​6.25, dry matter basis (DM)), ≤10% moisture, ≤2% ash (DM), ≤1.5% crude fiber (DM) and a variable percentage of residual starch and lipids. After rehydration, vital gluten regains its intrinsic functionality and forms a hydrated gluten network. This network has the ability to retain the gas produced during dough fermentation and it stabilizes the sponge-like structure of wheat bread crumb (Neumann, Kniel and Wassermann, 1997; Ortolan, Corrêa, da Cunha and Steel, 2017). Vital gluten is most commonly applied as an enhancer for weak flours to obtain improved dough strength, higher mixing tolerance, better gas holding capacity, higher baking volumes, and regular textural properties (Ortolan and Steel, 2017; Scherf et al., 2016). Vital gluten samples, even if they are from the same manufacturer, were often reported to have different qualities. Within the scope of this work, we defined vital gluten quality as good, if the addition of that sample resulted in a high bread volume in the baking experiments. One possible reason for different vital gluten qualities could be the drying process, as it is considered the most critical step in gluten production (Wadhawan and Bushuk, 1989; Weegels et al., 1994). Excessive heat during the drying process led to a loss of functional properties, “de-vitalized” vital gluten, and resulted in a reduced baking quality (Schofield et al., 1983). The prediction of vital gluten quality is difficult, because it has so far been unknown which target parameter(s) need(s) to be determined. Baking experiments are usually considered as the “golden standard” to identify the quality of vital gluten and used, e.g., by gluten producers and large bakeries. However, this test is time-consuming and labor-intensive, because it takes about 90 ​min from dough preparation to the final bread, depending on the procedure, plus an additional 30 ​min for evaluation (total time: 120 ​min).

This paper evaluates three possible approaches for a quick and reliable vital gluten quality assessment as alternative to baking experiments. These approaches were already successfully used to predict the baking quality of wheat flour, but to the best of our knowledge, this study is the first to investigate whether these three methods are also suitable to predict the quality of vital gluten. First, the GLIA/GLUT ratio could be an approach to determine gluten quality (Kieffer et al., 1998; Thanhaeuser et al., 2014). The procedure requires about 300 ​min considering weighing, extraction, analysis and data evaluation, depending on the fraction. It has no time advantage compared to the baking experiment, but it provides valuable information on the protein composition. Glutenin polymers contribute directly to the development of a gluten network by forming intermolecular disulfide bonds. Gliadins play an indirect role by weakening the interaction of the glutenin polymers due to the increase in entanglement spacing (Singh and MacRitchie, 2001; Delcour et al., 2012). Both gliadins and glutenins are considered to influence dough properties and need to be present in a balanced ratio to ensure good breadmaking performance (Goesaert et al., 2005). Second, the gluten aggregation test is considered as a tool to predict vital gluten quality. During the test, a suspension of vital gluten and water (ratio of approx. 2:1) is analyzed for its aggregation behavior. The input of mechanical energy via a rotating paddle leads to an increase in the consistency of the slurry up to a maximum value. This value represents the state when the gluten network is fully formed. As mechanical energy is continuously applied after reaching this state, the gluten network is destroyed, which results in a softening of the slurry consistency. Gluten aggregation parameters already showed promising correlations with quality-related gluten protein fractions of wheat flour (Marti et al., 2015) and were able to classify wheat according to dough stability (Malegori et al., 2018). Third, the microscale extension test, which provides information about the extensibility and the resistance to extension of each vital gluten sample, could be a possible alternative (Scherf et al., 2016). Both extensibility and resistance depend on the strength of the gluten network. While glutenin polymers contribute to dough strength and elasticity, gliadins provide viscosity and serve as plasticizers for the dough (Belton, 1999). All three approaches investigated require less time, labor, and testing material than baking experiments. In this investigation, we applied the three approaches (GLIA/GLUT ratio, gluten aggregation test, and microscale extension test) as well as microbaking tests to 46 vital gluten samples. To assess the ability of the three alternative approaches to replace baking experiments in predicting the quality of vital gluten, correlations between microbaking tests and each of the three approaches were calculated.

## 2 Materials and methods

### 2.1 Material

Vital gluten samples (G1-G46) were obtained from six different suppliers. Wheat flour type 1050 (Walzmühle, Horb-Altheim, Germany), sunflower seeds (Walz-Mühle, Horb-Altheim, Germany), soy flour (Zimmermann, Neu-Ulm, Germany), sesame (Seeberger, Ulm, Germany), rye sourdough (Böcker GmbH & Co. KG, Minden, Germany), lupine shots (Rapunzel Naturkost, Legau, Germany), linseeds (Walz-Mühle, Horb-Altheim, Germany), salt (Merck KGaA, Darmstadt, Germany) and roasted malt flour (Walz-Mühle, Horb-Altheim, Germany) were used to produce baking mixture A. Ireks GmbH (Kulmbach, Germany) kindly donated baking mixture B, a typical protein bread mixture. Brauerei Wieninger (Teisendorf, Germany) provided the yeast. All chemicals used were of analytical or higher quality and were purchased from either Merck KGaA (Darmstadt, Germany) or Sigma-Aldrich (Steinheim, Germany). Prof. Dr. Koehler, Chairman of the Working Group for Prolamin Analysis and Toxicity provided the reference material Prolamin Working Group (PWG)-gliadin (van Eckert et al., 2006).

### 2.2 Microbaking tests to determine the functionality of vital gluten samples

The external conditions remained constant for all tests (temperature 22 ​± ​2 ​°C, relative humidity ​≥ ​60%). All determinations were performed in triplicate. The first microbaking test was based on a recipe of 7.5 ​g baking mixture A (22.94% soy flour, 22.94% lupine shots, 18.35% linseeds, 11.47% sunflower seeds, 9.17% wheat flour type 1050, 6.88% rye sourdough, 4.59% sesame, 2.52% salt and 1.15% roasted malt flour), 2.5 ​g of one of the vital gluten samples G1-G46 each, 0.25 ​g yeast and 5.5 ​ml water (recipe A). The second recipe consisted of 7.5 ​g baking mixture B, 2.5 ​g of one of the vital gluten samples G1-G46 each, 0.25 ​g yeast and 7.5 ​ml water (recipe B). The exact composition of baking mixture B was unknown, but it was included for practical reasons, because it is a standard mixture commonly applied for high-protein breads. In both recipes all ingredients were kneaded for 8 ​min ​at 30 ​°C in a farinograph-E (Brabender, Duisburg, Germany). The dough was manually moulded and weighed about 13 ​g in total. Then, the dough piece was placed in a water-saturated proofer to rest for 20 ​min ​at 30 ​°C. Finally, the dough went through a fully-automated baking-line, consisting of a proofing chamber of 30 ​°C in which it was left to rest for 40 ​min as well as an oven in which it underwent a baking procedure for 10 ​min with the temperature increasing from 185 ​°C to 255 ​°C (Schaffarczyk et al., 2016). The volume of the resulting bread rolls was determined by a laser-based device (VolScan Profiler, Stable Micro Systems, Godalming, U.K.) after a 2 ​h cooling period. Afterwards, the specific volume (bread volume divided by dough weight) was calculated to compensate for, e.g., dough losses, which can occur during dough preparation and handling.

### 2.3 Determination of gluten protein composition by stepwise extraction and gel permeation high-performance liquid chromatography (GP-HPLC)

All vital gluten samples were extracted in triplicate by the modified Osborne fractionation (Schopf and Scherf, 2018) and analyzed by GP-HPLC. The process starts by mixing 20 ​mg of vital gluten samples G1-G46 with glass beads and extraction with 60% (v/v) aqueous ethanol (3 ​× ​1.5 ​ml) to obtain the gliadins. Next, glutenins were extracted by an extraction solution of 50% (v/v) propan-1-ol, 0.05 ​mol/l Tris-HCl (pH 7.5), 2 ​mol/l (w/v) urea and 1% (w/v) dithiothreitol (DTT) (3 ​× ​1.5 ​ml, under nitrogen atmosphere). The method contains three steps: 2 ​min vortex mixing, 10 ​min magnetic stirring at 22 ​°C for gliadins or 30 ​min stirring at 60 ​°C for glutenins, and 25 ​min centrifugation at 4600×*g* at 22 ​°C. The supernatants were combined and filled up to 5 ​ml with the corresponding extraction solution. An HPLC instrument from Hitachi Merck (VWR, Darmstadt, Germany) with a BioSep SEC-s3000 column (4.6 ​× ​300 ​mm, Phenomenex, Aschaffenburg, Germany) and the software LaChrom Elite (Version 3.1.1) was used for the analysis of the protein fractions. The instrument was set to: column temperature; 22 ​°C, flow rate; 0.3 ​ml/min, injection volume; 20 ​μl, solvent; water/trifluoroacetic acid (TFA, 999/1, v/v) (A) and acetonitrile/TFA (999/1, v/v) (B), isocratic 50% A/50% B. PWG-gliadin (11.6–46.6 ​μg were dissolved in 60% (v/v) ethanol) was used as calibration material (van Eckert et al., 2006) to calculate the protein content. The obtained peaks had a retention time of 6.0–13.0 ​min. The molecular weight was between 80 ​000 and 100 ​000 for high-molecular-weight (HMW), 50 ​000 and 80 ​000 for medium-molecular-weight (MMW) and 30 ​000 and 50 ​000 for low-molecular-weight (LMW) gluten proteins. HMW-gliadins occurred from 6.0 to 8.1 ​min, MMW-gliadins from 8.1 to 9.1 ​min and LMW-gliadins from 9.1 to 13.0 ​min. HMW-glutenins were observed from 6.0 to 7.4 ​min, MMW-glutenins from 7.4 to 8.6 ​min and LMW-glutenins from 8.6 to 13.0 ​min.

### 2.4 Determination of gluten aggregation behavior

The aggregation behavior of each vital gluten sample was measured in triplicate with a GlutoPeak instrument (Brabender, Duisburg, Germany) by applying the method described in the Technical Note of Gall et al. (2017). Vital gluten (2.10 ​g) was suspended in 4.41 ​g distilled water in the stainless-steel sample cup. The instrument temperature was set to 36 ​°C. The speed profile for the rotating paddle was defined in the software (GlutoPeak, version 2.2.6) and set to 500 ​rpm for 1 ​min, 0 ​rpm for 2 ​min and 3300 ​rpm for 10 ​min. The software provided the curve profile (gluten aggregation over time) as well as the maximum torque (BEM) expressed in Brabender units (BU) and the peak maximum time (PMT) expressed in seconds.

### 2.5 Microscale extension tests of hydrated vital gluten

The sample preparation procedure and the microscale extension test of hydrated gluten were described in detail by Scherf et al. (2016). The force-distance curves of each vital gluten sample were carried out in triplicate from four different experiments (n ​= ​3 ​× ​4 ​= ​12). Therefore, three steps were necessary: hydration of vital gluten, centrifugation and microscale extension. For hydration, 1.5 ​g vital gluten were mixed in a 50 ​ml beaker with 5 ​ml of a salt solution (2% NaCl) until no dry powder was left. After an incubation period of 5 ​min the hydrated vital gluten was placed between a specially notched and a smooth Teflon mould and centrifuged in cylindrical centrifuge inserts (Heraeus Labofuge 400 ​R, Thermo Fisher Scientific, Osterode, Germany) for 10 ​min ​at 3060×*g* and 22 ​°C. The preformed gluten strands were pressed between a sufficiently oiled trapezoidal ribbed and a smooth Teflon plate and protruding vital gluten parts were removed. The gluten strand was placed on the measuring device after an incubation time of 15 ​min. The SMS/Kieffer Dough and Gluten Extensibility Rig fitted to a TA.XT plus Texture Analyzer (Stable Micro Systems, Godalming, U.K.) was used with a 5 ​kg load cell and the software Exponent version 6.1.7. The following parameters were set: test mode: extension, pre-test speed: 2.0 ​mm/s, test speed: 3.3 ​mm/s, post-test speed: 20.0 ​mm/s, rupture distance: 4.0 ​mm, distance: 150 ​mm, force: 0.049 ​N, time: 5 ​s, trigger type: auto, trigger force: 0.049 ​N, break detect: rate, break sensitivity: 0.020 ​N.

### 2.6 Statistical data analysis

One-way analysis of variance (one-way ANOVA) was applied to determine significant differences between vital gluten samples (SigmaPlot 11, Systat Software, San Jose, USA). These differences were selected with Tukey's test at a significance level of p ​< ​0.05. The specific volumes of both microbaking tests were used to perform a hierarchical cluster analysis to classify the 46 vital gluten samples into the quality classes good, medium and poor using Origin 2019 (OriginLab Corporation, Northampton, USA). The means for all variables were calculated for each cluster by applying the cluster analysis. Then, the Euclidean distance to the cluster means was determined for each vital gluten sample and similar values based on the sum of the squared distances were assigned to one cluster. The peak30 (area from 15 ​s before the PMT to 15 ​s after the PMT) and peak180 (area from 180 ​s after the start of the measurement to 15 ​s after the PMT) were calculated manually to get more details about the profile of the GlutoPeak curve. Furthermore, the curve was fitted with the Chesler-Cram Peak Function (CCE) and the resulting parameters were assessed in a correlation matrix. The equation of the fit is as follows:

(1) $${\text{y}=\text{y}}_{\text{0}}+\text{A}\left[\text{exp}-\left({\left({\text{x}-\text{x}}_{\text{c}1}\right)}^{2}/2\text{w}\right)+\text{B}\left(1-0.5\left(1-\text{tanh}\left({\text{k}}_{2}\left({\text{x}-\text{x}}_{\text{c}2}\right)\right)\right)\right)\text{exp}\left({-0.5\text{k}}_{3}\left(\left|{\text{x}-\text{x}}_{\text{c}3}\right|+\left({\text{x}-\text{x}}_{\text{c}3}\right)\right)\right)\right]$$

where y_0_ is the offset, x_c1_ is the first center, A is the first amplitude, w is the half width, k_2_ is the first unknown, x_c2_ is the second center, B is the second amplitude, k_3_ is the second unknown, and x_c3_ is the third center.

Origin 2019 was used to evaluate the suitability of the three approaches (GLIA/GLUT ratio, gluten aggregation test and microscale extension test) to predict vital gluten quality. Spearman correlations were applied to relate the respective parameters with the specific volumes of baking mixture A and B at a significance level of p ​< ​0.05.

### 2.7 Development of a scoring system

A scoring system was developed using those parameters of the gluten aggregation test and the microscale extension test that showed a significant correlation to the specific volumes of the microbaking tests (Spearman's correlation coefficients (r_S_)). First, value ranges for the different quality classes were defined for each parameter (Table S1). For this purpose, the 25% and the 75% quantiles of the “medium” group, as defined by the hierarchical cluster analysis, were calculated using Origin 2019 to ensure that the majority of the vital gluten samples with medium quality will be correctly assigned to the “medium” group. The measured values resulting from the gluten aggregation test and microscale extension test were then matched to the pre-defined parameter ranges and corresponding points were allocated. Values that belonged to the good quality class were attributed 20 points, those of the medium quality class 10 points, and those of the poor quality class 0 points. Those points were then multiplied by the respective correlation coefficient to account for the different accuracy to predict the specific volume of vital gluten. For example, G16 received 20 points for PMT, which were multiplied by the correlation coefficient of 0.53 (r_s_ of PMT) resulting in a weighted value of 10.6. The weighted values of all parameters were summed up and assigned based on the following classification: vital gluten samples that reached a total greater than 80 points were classified as good, greater than 50 as medium, and less than 50 as poor.

## 3 Results & discussion

### 3.1 Classification of vital gluten samples into quality classes

**Fig. 1:**
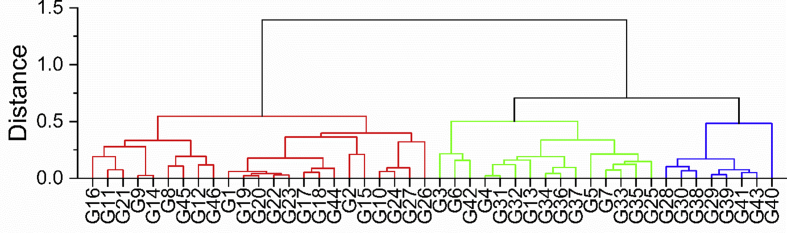
Hierarchical cluster analysis based on the specific volumes of both baking mixtures A and B. The division was made into the three quality classes “good”, “medium” and “poor”. Twenty-three vital gluten samples were classified as good (left cluster, red), 15 as medium (middle cluster, green) and eight as poor (right cluster, blue). (For interpretation of the references to colour in this figure legend, the reader is referred to the Web version of this article.)

As baking experiments are the “golden standard” for evaluating the quality of vital gluten (Gabriel et al., 2017), the vital gluten samples G1-G46 were classified based on the specific volumes of two microbaking tests using baking mixture A and B (Table 1). The correlation between both microbaking tests was significant and very high (r_S_ ​= ​0.893, p ​< ​0.001). The breads made from baking mixture A had specific volumes from 1.6 ​ml/g (G40) to 3.5 ​ml/g (G2). The breads made from baking mixture B generally resulted in lower specific volumes from 1.1 ​ml/g (G40) to 3.0 ​ml/g (G26) and we assume that the lower specific volumes were caused by the higher water addition. The comparatively complex recipes were chosen, because preliminary experiments using 7.5 ​g of a weak wheat flour as a base and 2.5 ​g of vital gluten gave very similar results for all samples and the resulting specific volumes hardly showed any significant differences. In contrast, the specific volumes differed significantly among vital gluten samples within one microbaking test using either baking mixture. Based on the specific volumes of both recipes, a hierarchical cluster analysis was performed, resulting in three different cluster types (good, medium, and poor) (Fig. 1). Twenty-three vital gluten samples were classified as quality class “good”, 15 as “medium” and the remaining 8 as “poor” (Table 1). Breads made with poor vital gluten had a firmer and moister crumb compared to breads made with medium or good vital gluten. Vital gluten samples of medium and good quality resulted in a regular crumb structure, but differed in their specific volumes, which were higher for the good quality breads. Measurements of rheological properties were beyond the scope of this work, but further studies could be interesting to provide some more in-depth insights.

**Table 1:** Specific volumes [ml/g] of microbaking tests (recipe A and B) using vital gluten (VG) samples G1-G46 as well as their classification group (good, medium or poor) resulting from the hierarchical cluster analysis.

VG	Specific volume A		Specific volume B		Class
	Mean [ml/g]^a^	RSD	Mean [ml/g]^a^	RSD	
G1	3.2^jklmn^	5.9	2.5^ijklmno^	2.7	good
G2	3.5^n^	5.9	2.7^lmnop^	3.1	good
G3	3.1^hijklmn^	3.1	1.7^def^	7.2	medium
G4	2.6^bcdef^	4.8	1.3^abcd^	6.2	medium
G5	2.7^defghij^	7.7	1.5^bcde^	3.8	medium
G6	2.9^fghijklm^	6.5	1.7^ef^	2.0	medium
G7	2.8^efghijk^	1.6	1.3^abcde^	0.6	medium
G8	2.8^efghijk^	2.5	2.3^ghijk^	8.8	good
G9	2.9^fghijkm^	2.8	2.0^fg^	6.2	good
G10	3.0^hijklmn^	4.3	2.9^op^	5.8	good
G11	3.0^hijklmn^	8.2	2.2^gh^	8.3	good
G12	2.9^efghijklm^	3.6	2.5^hijklm^	9.0	good
G13	2.6^cdefgh^	8.5	1.2^ab^	7.1	medium
G14	2.9^fghijklmn^	3.3	2.0^fg^	6.7	good
G15	3.4^lmn^	4.2	2.6^ijklmno^	6.5	good
G16	3.2^klmn^	2.6	2.3^ghi^	9.4	good
G17	2.9^fghijklm^	5.0	2.5^ijklmn^	4.7	good
G18	3.0^ghijklmn^	5.3	2.6^jklmno^	3.7	good
G19	3.2^jklmn^	3.7	2.6^klmno^	9.8	good
G20	3.2^jklmn^	5.7	2.6^jklmno^	7.1	good
G21	3.1^ijklmn^	3.6	2.2^ghij^	1.8	good
G22	3.1^jklmn^	5.8	2.5^ijklmno^	3.5	good
G23	3.1^jklmn^	3.9	2.6^jklmno^	6.7	good
G24	3.1^hijklmn^	3.0	2.9^nop^	6.1	good
G25	2.8^efghijkl^	7.0	1.2^abc^	4.5	medium
G26	3.4^mn^	9.6	3.0^p^	3.2	good
G27	3.1^jklmn^	2.8	2.8^mnop^	4.7	good
G28	2.2^bcd^	8.3	1.2^abc^	4.5	poor
G29	2.0^ab^	8.3	1.1^a^	2.7	poor
G30	2.1^abc^	7.9	1.2^ab^	4.3	poor
G31	2.6^cdefghi^	6.0	1.3^abc^	2.4	medium
G32	2.4^bcdefg^	8.6	1.4^abcde^	4.9	medium
G33	2.7^defghijk^	5.0	1.4^abcde^	4.5	medium
G34	2.4^bcdef^	5.0	1.2^abc^	8.6	medium
G35	2.9^fghijklm^	4.3	1.4^abcde^	4.9	medium
G36	2.4^bcdef^	8.5	1.3^abc^	1.3	medium
G37	2.3^bcde^	3.9	1.2^abc^	2.6	medium
G38	2.2^bc^	3.1	1.1^ab^	0.5	poor
G39	2.0^ab^	6.9	1.1^ab^	2.7	poor
G40	1.6^a^	4.0	1.1^a^	0.2	poor
G41	2.0^ab^	6.5	1.1^ab^	5.9	poor
G42	2.8^efghijkl^	5.9	1.6^cde^	4.2	medium
G43	2.1^abc^	3.3	1.1^ab^	2.0	poor
G44	3.0^hijklmn^	3.9	2.6^ijklmno^	4.9	good
G45	2.9^efghijklm^	2.3	2.2^ghij^	1.6	good
G46	2.8^efghijkl^	2.0	2.3^ghijkl^	4.2	good

a Mean value of n = 3; RSD: relative standard deviation; Mean values associated with different small superscript letters indicate significant differences between vital gluten samples within one experimental setup (one-way ANOVA, Tukey's test, p < 0.05).

### 3.2 Determination of gluten protein composition by GP-HPLC

**Fig. 2:**
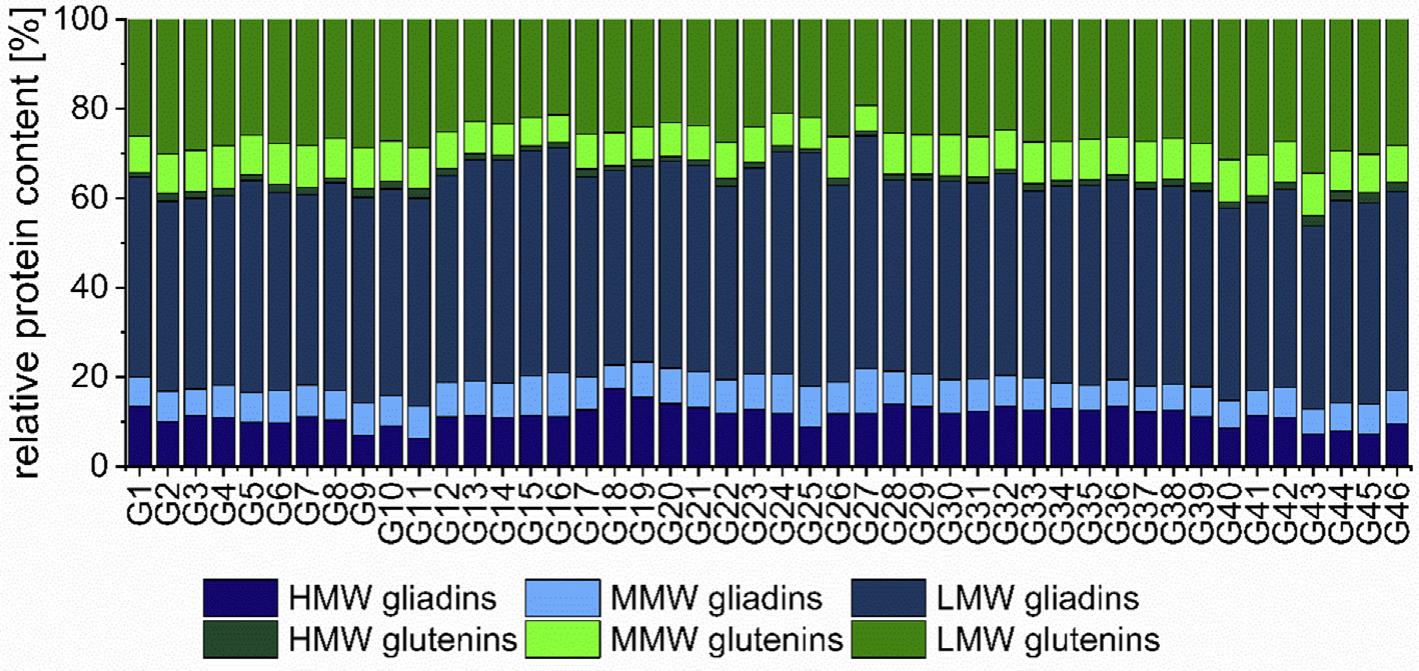
Relative protein content of vital gluten samples G1-G46 determined by GP-HPLC [%]. Data is presented as mean value (n ​= ​3) of relative high-molecular-weight (HMW)-, medium-molecular-weight (MMW)- and low-molecular-weight (LMW)-gliadins and HMW-, MMW- and LMW-glutenins.

The protein distribution of the vital gluten samples G1-G46 was determined according to Grosch and Wieser (1999) and is shown in Fig. 2. The proportions ranged from 6.2% (G11) to 17.5% (G18) for HMW-gliadins, 5.2% (G18) to 10.1% (G27) for MMW-gliadins and 41.0% (G43) to 52.1% (G25) for LMW-gliadins. In total, gliadins made up between 53.9% (G43) and 73.9% (G27). For glutenins, the relative protein distribution was 0.8% (G1) to 2.4% (G45) for HMW-glutenins, 5.8% (G27) to 9.5% (G4) for MMW-glutenins and 19.3% (G27) to 34.5% (G43) for LMW-glutenins. Overall, glutenins ranged from 26.1% (G27) to 46.1% (G43). The resulting GLIA/GLUT ratio was between 1.2 (G43) and 2.8 (G27). To obtain a general view, the mean values (MV) of protein-related parameters were determined for each cluster (Table S1). It revealed that the MV of MMW-, LMW- and total gliadins of lower quality vital gluten were lower compared to those of higher quality gluten. In contrast, the MV of MMW-, LMW- and total glutenins were higher. Additionally, the GLIA/GLUT ratio was highest for the good quality, followed by the medium quality and it was lowest for the poor quality. The proportions of HMW-gliadins, HMW-glutenins and total gluten were similar between the quality classes. In general, dough properties are influenced by the content and composition of gliadins and glutenins. While gliadins are responsible for higher viscosity and thus for dough extensibility, glutenins are associated with dough elasticity and therefore with dough strength (Delcour et al., 2012). A high GLIA/GLUT ratio ensures high viscosity and leads to low resistance to extension as well as high elasticity (Marti et al., 2015; Wieser and Kieffer, 2001). Therefore a balance between gliadins and glutenins is necessary to obtain a high specific volume. The highest specific volume was achieved by vital gluten sample G2 with a GLIA/GLUT ratio of 1.5 for baking mixture A and by vital gluten G27 with a GLIA/GLUT ratio of 1.7 for baking mixture B.

### 3.3 Gluten aggregation test

**Fig. 3:**
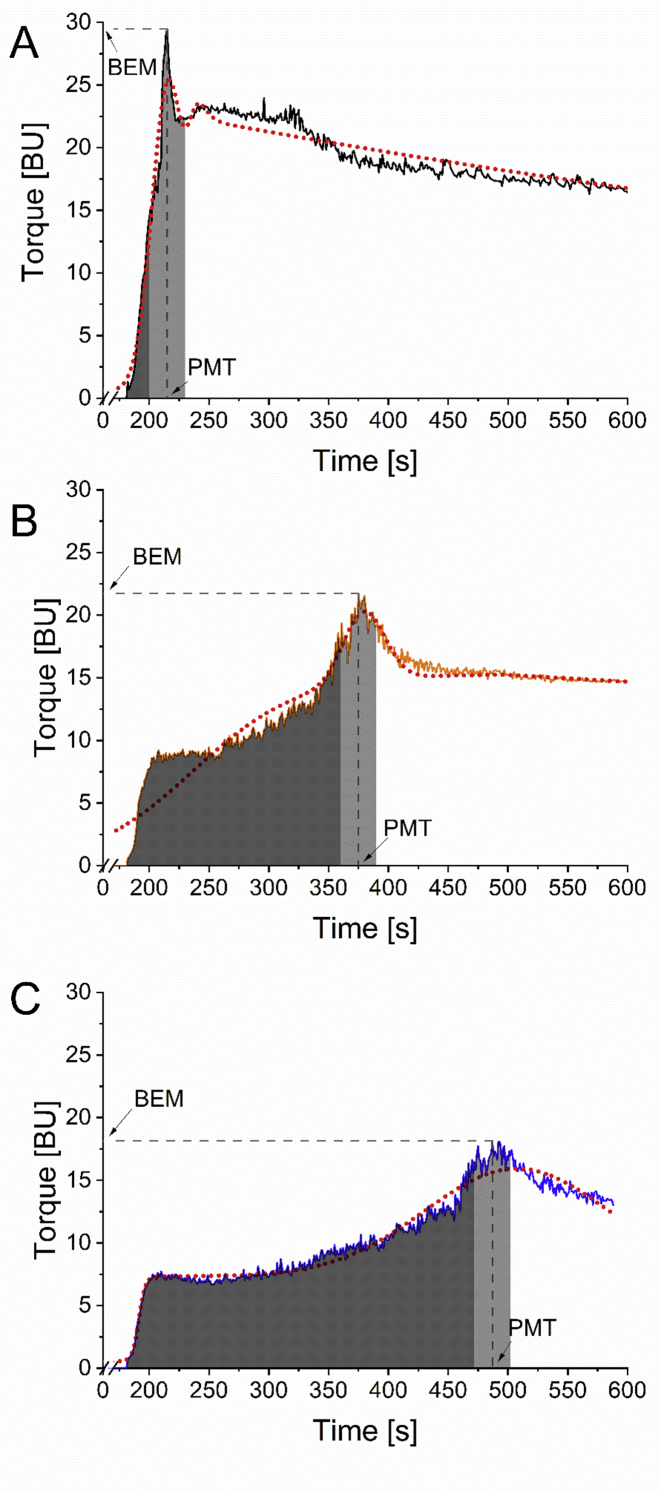
Exemplary curves of vital gluten samples G18 with good quality (A), G6 with medium quality (B) and G41 with poor quality (C) determined by GlutoPeak and the resulting parameters: Peak maximum time (PMT) expressed in [s], maximum torque (BEM) expressed in Brabender units [BU], Peak30 represented by the light grey area and Peak180 represented by the sum of the dark and light grey areas.

The gluten aggregation behavior of all 46 vital gluten samples was analyzed using the GlutoPeak instrument. An exemplary curve of a representative vital gluten sample of each quality class is shown in Fig. 3. A high BEM value and a fast PMT were characteristic of vital gluten samples which were classified as good (Fig. 3A). In comparison, the curve profile of vital gluten samples of medium quality showed a later PMT and a lower BEM (Fig. 3B). Vital gluten samples of poor quality had a late PMT and a low BEM (Fig. 3C). Overall, the PMT ranged between 213.7 ​s (G18) and 486.7 ​s (G41) and the BEM was between 23.0 BU (G37) and 33.3 BU (G25) (Table S2). Beside those parameters provided by the GlutoPeak software, the peak30, the peak180 and their ratio were manually calculated for each vital gluten sample in order to characterize the curve in more detail. Peak30 was between 380.1 area units (AU) (G37) and 724.1 AU (G25), peak180 was between 647.7 AU (G20) and 3772.5 AU (G25) and the ratio peak30/peak180 was 0.2–0.9. The comparison of the MV from each quality class showed that peak30 remained similar and did not show large differences between good and poor quality. Peak180 increased and the area ratio decreased with decreasing vital gluten quality. A comparison of the peak180 values was valuable for defining the curve characteristics and thus supported the quality assessment. A CCE fit was used to approximate the actual curve as it had a high coefficient of determination (R^2^ higher than 0.9). The parameters resulting from the CCE equation were included in the correlation analysis (Table 2). The MV of amplitude A and the halfwidth w were higher for vital gluten samples classified as good compared to samples of medium or poor quality (Table S1). All other parameters were similar among the clusters. Previous studies showed promising correlations for wheat flours between the parameters determined by the gluten aggregation test and the baking performance (Bouachra, Begemann, Aarab, & Hüsken 2017; Marti, Augst, Cox and Koehler, 2015). Weak flours were characterized by a rapid formation of the gluten network, followed by a fast degradation, as opposed to strong flours that took more time to build up the gluten network, but remained more stable (Goldstein et al., 2010). In contrast to wheat flour, the vital gluten sample was not continuously exposed to mechanical stress during the measurement. There was a 1 ​min pre-shearing step at 500 ​rpm during which the hydration of the vital gluten sample took place. Then, the vital gluten sample was left to rest for 2 ​min to allow relaxation prior to the actual measurement (Gall et al., 2017). Vital gluten contains only small amounts of starch compared to wheat flour. This facilitates the formation of a gluten network, as there is hardly any steric hindrance from starch particles. This could be the reason why vital gluten samples of good quality displayed earlier and sharper peaks compared to vital gluten samples of poor quality.

**Table 2:** Correlation coefficients (r_S_) and corresponding level of significance (p-value) between the specific volume (recipe A and B) and the parameters of the three approaches (GlutoPeak: peak maximum time (PMT), torque (BEM), area 15 ​s before and after PMT (Peak30), area from 180 ​s after the beginning of the measurement to 15 ​s after the PMT (Peak180), Peak30/Peak180 and CCE equation parameters (y0, xc1, A, w, k2, xc2, B, k3 and xc3); microscale extension test: maximum resistance to extension (R_max_), distance at maximum resistance to extension (E_Rmax_), area under the curve (R_max_), distance at maximum extensibility (E_max_), area under the total curve (A_max_) and the ratio E_max_/R_max_ and gluten protein composition: high-molecular-weight (HMW)-, medium-molecular-weight (MMW)-, low-molecular-weight (LMW)-gliadins and glutenins and gliadin-to-glutenin (GLIA/GLUT) ratio.

	Specific volume A		Specific volume B	
	r_S_	p-value^a^	r_S_	p-value^a^
PMT	−0.545	≤0.001	−0.515	≤0.001
BEM	0.594	≤0.001	0.524	≤0.001
Peak30	0.212	n.s.	0.222	n.s.
Peak180	−0.437	0.002	−0.393	0.007
Peak30/Peak180	0.540	≤0.001	0.484	≤0.001
y0	0.059	n.s.	−0.040	n.s.
xc1	−0.560	≤0.001	−0.525	≤0.001
A	0.166	n.s.	0.143	n.s.
W	−0.347	0.018	−0.292	0.049
k2	0.139	n.s.	0.199	n.s.
xc2	0.352	0.016	0.349	0.017
B	−0.257	n.s.	−0.255	0.087
k3	−0.218	n.s.	−0.146	n.s.
xc3	−0.506	≤0.001	−0.509	≤0.001
E_Rmax_	0.442	0.002	0.521	≤0.001
R_max_	0.150	n.s.	0.066	n.s.
A_Rmax_	0.398	0.002	0.396	0.002
E_max_	0.516	≤0.001	0.614	≤0.001
A_max_	0.493	≤0.001	0.499	≤0.001
E_Rmax_/R_max_	0.235	n.s.	0.370	n.s.
HMW gliadins	0.026	n.s.	0.029	n.s.
MMW gliadins	0.433	0.003	0.390	0.007
LMW gliadins	0.370	0.011	0.374	0.010
Gliadinsrowhead	0.327	0.026	0.301	0.042
HMW glutenins	−0.033	n.s.	0.061	n.s.
MMW glutenins	−0.507	≤0.001	−0.487	≤0.001
LMW glutenins	−0.309	0.037	−0.297	0.045
Glutenins	−0.327	0.026	−0.301	0.042
GLIA/GLUT ratio	0.327	0.026	0.301	0.042

a p ​> ​0.05: not significant (n.s.), p ​≤ ​0.05: significant; p ​≤ ​0.001 highly significant.

### 3.4 Microscale extension test

**Fig. 4:**
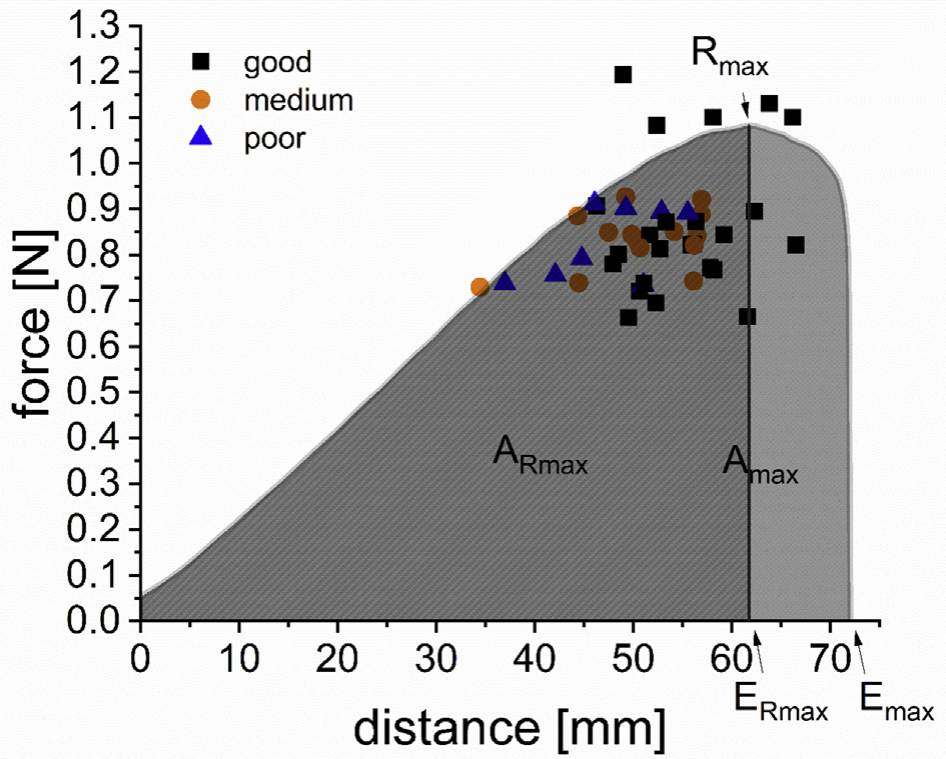
Exemplary force-distance curve of rehydrated vital gluten sample G10 and the resulting parameters: maximum resistance to extension (R_max_ in [N]), corresponding distance at maximum resistance to extension (E_Rmax_ in [mm]), corresponding area under the curve (A_Rmax_ in [mJ], dark grey area), distance at maximum extensibility (E_max_ in [mm]), and corresponding area under the total curve (A_max_ in [mJ], dark and light grey areas). Force-distance scatter plot of vital gluten samples G1-G46. Vital gluten samples are classified according to their quality: black rectangles (good), orange circles (medium) and blue triangles (poor). (For interpretation of the references to colour in this figure legend, the reader is referred to the Web version of this article.)

The force-distance curves of all vital gluten samples were recorded (Fig. 4). The maximum resistance to extension R_max_ (0.7 ​N (G46) to 1.2 ​N (G26)), the corresponding distance at maximum resistance to extension E_Rmax_ (34.5 ​mm (G13) to 66.5 ​mm (G22)), the corresponding area under the curve A_Rmax_ (14.9 ​mJ (G13) to 42.4 ​mJ (G24)), the distance at maximum extensibility E_max_ (42.2 (G13) to 78.1 (G22)), the corresponding area under the total curve A_max_ (19.9 (G13) to 54.9 (G24)) and the ratio E_max_/R_max_ (41.0 (G26) to 92.6 (G27)) were provided by the software (Table S2). The MV of all parameters were calculated for each cluster (Table S1). While R_max_ was independent of vital gluten quality, the other parameters showed a lower MV for vital gluten samples with decreasing quality. Similar results were already reported (Thanhaeuser et al., 2014; Kieffer et al., 1998).

### 3.5 Correlation matrix

The parameters of the three approaches were correlated with the specific volumes of the two microbaking tests (Table 2). The GLIA/GLUT ratio was weakly correlated. MMW-, LMW-gliadins and total gliadins, as well as, MMW-, LMW-glutenins and total glutenins were significantly correlated, but the correlation coefficients were weak. Therefore, the approach of determining the GLIA/GLUT ratio was not sufficiently suitable to predict vital gluten quality as defined by its functionality, i.e. high volume, in the microbaking tests used here. The parameters of the GlutoPeak test basically showed good significant correlations to the results of the microbaking tests. The PMT, the BEM, the peak180 and the peak30/peak180 ratio could be possible predictors for breadmaking performance. The gluten aggregation test has already been considered as an alternative prediction tool for wheat flour quality (Malegori et al., 2018; Marti et al., 2015; Zawieja et al., 2020). Sissons (2016) indicated that the PMT was the best parameter for predicting gluten strength for durum wheat and was able to separate strong and weak dough samples. A previous study of Bouachra et al. (2017) showed that a linear model based on the combination of the crude protein content and the GlutoPeak parameters was able to predict the loaf volume of 64% of independent data correctly. In addition, the microscale extension test could be suitable to predict vital gluten quality. The relationship between the parameters of the microscale extension test and the specific volume was significant except for R_max_. E_Rmax_, A_Rmax_, E_max_ and A_max_ showed correlation coefficients between 0.4 and 0.5.

### 3.6 Evaluation of the scoring system

The combination of the gluten aggregation test and the microscale extension test was evaluated for its predictive value regarding bread volumes using a scoring system (Table S3). Considering all significant parameters, the scoring system was able to predict 65.2% of vital gluten samples correctly into their quality class. By using only the significant parameters of the gluten aggregation test, still 63.0% of vital gluten samples were assigned correctly, while 52.2% of vital gluten samples would be allocated into the correct quality class only considering parameters of the microscale extension test. Using a combination of both, the result of 65.2% was a good indication and we considered this approach to be feasible to determine the quality of vital gluten. The percentage of correct assignments could actually be higher, but there were some incorrect assignments of vital gluten samples. This might be caused by several possibilites. On the one hand, the cluster formation had a huge impact on the assignment of the vital gluten samples into their quality classes. The classification was based on the specific volumes of both microbaking tests to account for differences caused by the composition of the recipes. For example, vital gluten sample G3 showed a score of 85.8 determined by the parameters of the gluten aggregation test and of the microscale extension test, resulting in a prediction of good quality. G3 reached a specific volume of 3.1 ​ml/g for recipe A, but only 1.7 ​ml/g for recipe B. Considering only recipe A, G3 would be assigned as good. Since both recipes A and B were used for the classification the actual result was more accurate and G3 received a correct rating of medium, because of its low bread volume in recipe B. On the other hand, the vital gluten samples showed structural similarities and the transition from poor to medium and from medium to good was very close. For this reason, the error propability was comparatively high. For example, the score of vital gluten G46 was 75.6, which led to a false identification as medium quality, but actually it should have been good according the hierarchical clustering. For a more detailed evaluation of the performance and prediction accuracy of the scoring system, further work will include more and new vital gluten samples.

## 4 Conclusion

This study showed that protein-related parameters, such as the GLIA/GLUT ratio, were not reliable enough to predict gluten quality, because the correlations to the volumes of the microbaking tests were weak. The gluten aggregation test and the microscale extension test were reliable methods to predict vital gluten quality for use in baking. Both methods need less effort in terms of material, time and human resources compared to baking experiments. The time saving compared to the microbaking test was 100 ​min for the gluten aggregation test and 78 ​min for the microscale extension test. Especially, BEM, PMT, Peak30/Peak180 ratio as well as E_Rmax_ can be suitable alternative quality predictors.

## Credit author statement

Katharina Scherf: Conceptualization; Funding acquisition; Project administration; Resources; Supervision; Writing - review & editing. Marina Schopf: Data curation; Formal analysis; Investigation; Methodology; Visualization; Writing - original draft.

## Declaration of competing interest

The authors declare that they have no known competing financial interests or personal relationships that could have appeared to influence the work reported in this paper.
