# Supplementary material for: Predicting vital wheat gluten quality using the gluten aggregation test and the microscale extension test
Source: Curr Res Food Sci. 2020 Dec 3;3:322–8. doi: 10.1016/j.crfs.2020.11.004 (PMC7749392; doi:10.1016/j.crfs.2020.11.004)
Supplement: Multimedia component 1 [file mmc1.pdf]

Predicting vital wheat gluten quality using the gluten aggregation test and the microscale extension test

Supplementary Material

Marina SCHOPF[1], Katharina Anne SCHERF[1, 2*]

[1]Leibniz-Institute for Food Systems Biology at the Technical University of Munich, Lise-Meitner-Strasse 34, 85354 Freising, Germany

[2]Department of Bioactive and Functional Food Chemistry, Institute of Applied Biosciences, Karlsruhe Institute of Technology (KIT), Adenauerring 20a, 76131 Karlsruhe

Table S1. Calculated mean values of the parameters from the microbaking tests A and B, the microscale extension test, the GlutoPeak test and the protein distribution within each cluster, as well as the 25% and 75% percentile of the medium quality class. Twenty-three vital gluten samples for the good, 15 for the medium and eight for the poor quality were taken into account.

	Unit	Mean value			P25	P75
		good	medium	poor	medium	
specific volume A	[ml/g]	3.1	2.7	2.0	-	-
specific volume B	[ml/g]	2.5	1.4	1.1	-	-
PMT	[s]	304.6	378.2	401.9	361.0	420.7
BEM	[BU]	27.7	26.4	25.4	25.0	43917,0
PMT • BEM	[BU•s]	8343.0	10008.6	10225.5	9025.0	10969.3
Peak30	[AU]	667.5	641.8	654.7	-	-
Peak180	[AU]	2020.5	2802.0	3108.0	2302.3	3271.8
Peak30/Peak180		0.4	0.2	0.2	0.2	0.3
y0		-6621.1	-2512.0	-8419.0	-	-
xc1		312.0	732.7	410.6	375.7	534.6
A		1122.4	34.8	6.5	-	-
w		543404.7	412441.6	1345.0	572.8	23395.1
k2		-1.7	-79565100	0.04	-	-
xc2		16.4	-98853800	-220.9	-	-
B		922.5	-137009000	1417.1	-	-
k3		0.6	0.7	0.6	-	-
xc3		-215.7	1434.1	796.3	412.6	555.4
E_{Rmax}	[mm]	55.2	51.0	47.3	47.5	56.5
R_{max}	[N]	0.9	0.9	0.8	-	-
$E_{Rmax} \cdot R_{max}$	[N•mm]	0.05	0.04	0.04	0.04	0.05
A_{Rmax}	[AU]	28.1	25.8	23.2	23.9	27.9
E_{max}	[mm]	66.0	59.7	55.3	56.1	65.3
A_{max}	[AU]	36.5	32.5	29.2	29.7	35.7
E_{max} / R_{max}	[mm/N]	66.1	60.1	57.2	-	-
HMW-gliadins	[mg/g]	94.5	94.7	93.9	-	-
MMW-gliadins	[mg/g]	64.1	56.5	54.0	-	-
LMW-gliadins	[mg/g]	390.2	371.3	357.6	-	-
Total gliadins	[mg/g]	548.8	522.5	505.5	-	-
HMW-glutenins	[mg/g]	12.5	11.4	12.5	-	-
MMW-glutenins	[mg/g]	65.9	71.6	75.9	-	-
LMW-glutenins	[mg/g]	211.8	217.2	235.7	-	-
Total glutenins	[mg/g]	290.2	300.2	324.1	-	-
Total gluten	[mg/g]	839.0	822.7	829.5	-	-
HMW-gliadins	[%]	11.2	11.6	11.3	-	-
MMW-gliadins	[%]	7.5	6.8	6.5	-	-
LMW-gliadins	[%]	46.3	44.9	43.1	-	-
Total gliadins	[%]	65.0	63.3	60.9	-	-
HMW-glutenins	[%]	1.5	1.4	1.5	-	-
MMW-glutenins	[%]	7.9	8.8	9.2	-	-
LMW-glutenins	[%]	25.5	26.5	28.4	-	-
Total glutenins	[%]	35.0	36.7	39.1	-	-
GLIA/GLUT ratio		1.9	1.7	1.6	-	-

Table S2. Comparison of 46 vital gluten (VG) samples G1-G46 based on rheological parameters of GlutoPeak and microscale extension tests. The values represent the means (n=3) for GlutoPeak and (n=12) for microscale extensions tests) with their corresponding relative standard deviation (RSD). Different small superscript letters refer to significant differences between the samples within each column (one-way ANOVA, Tukey's test, $p < 0.05$).

VG	PMT		BEM		peak30	peak180	peak30/ peak180	R_{max}		E_{Rmax}		A_{Rmax}		E_{max}		A_{max}		E_{Rmax}/R_{max}	
	[s][1]		[BU][1]		[AU]	[AU]		[N][2]		[mm][2]		[mJ][2]		[mm][2]		[mJ][2]			
	Mean	RSD	Mean	RSD				Mean	RSD	Mean	RSD	Mean	RSD	Mean	RSD	Mean	RSD	Mean[2]	RSD
G1	373.3^{klmno}	1.2	27.3^{bcdefg}	2.1	599.4	2491.5	0.2	1.1^{l}	4.7	$52.4^{defghijkl}$	6.5	32.9^{opq}	9.9	64.4^{fghijk}	4.7	44.4^{no}	9.1	48.4^{abc}	6.3
G2	371.7^{klmno}	1.8	$26.7^{abcdefg}$	2.2	570.7	2367.2	0.2	1.1^{l}	4.6	66.1^{pq}	6.1	42.2^{rs}	5.2	77.0^{no}	4.9	53.2^{pq}	6.9	$60.8^{cdefghi}$	8.8
G3	365.0^{klmn}	5.0	27.0^{bcdefg}	6.4	532.2	2209.2	0.2	0.9^{jk}	6.1	$56.9^{ijklmno}$	5.8	30.6^{mnop}	9.7	65.3^{ghijk}	5.7	37.6^{ijklm}	8.6	62.7^{ghijkl}	8.2
G4	433.0^{rs}	5.4	25.3^{abcde}	4.6	464.1	2583.6	0.2	0.7^{abcd}	7.6	$56.1^{ijklmno}$	6.5	$24.3^{defghijk}$	7.7	64.0^{fghij}	8.7	29.7^{cdefgh}	11.7	$76.0^{efghijk}$	11.9
G5	420.7^{pqrs}	1.6	$28.0^{cdefghi}$	3.6	608.9	3117.0	0.2	0.9^{hijk}	6.3	$56.9^{ijklmno}$	6.9	29.7^{lmno}	11.6	$66.8^{hijklmn}$	7.3	37.7^{jklmn}	15.0	64.1^{lm}	7.1
G6	376.3^{lmno}	0.7	27.3^{bcdefg}	2.1	585.0	2420.0	0.2	$0.8^{efghijk}$	3.9	$56.5^{ijklmno}$	8.1	27.9^{jklmno}	10.8	66.2^{hijkl}	9.9	35.4^{hijklm}	13.5	$67.2^{fghijkl}$	9.1
G7	418.0^{pqrs}	3.1	$26.3^{abcdefg}$	2.2	490.3	2583.7	0.2	0.8^{efgijk}	4.9	$50.4^{cdefghij}$	11.9	$24.7^{efghijkl}$	11.6	59.0^{defgh}	11.0	$31.1^{defghij}$	11.8	$59.5^{bcdefghi}$	16.0
G8	401.7^{nopqr}	1.5	26.0^{abcdef}	0.0	555.5	2662.5	0.2	0.8^{bcdef}	6.1	$57.8^{jklmnop}$	6.9	$25.7^{fghijklm}$	7.8	66.2^{hijkl}	5.9	$31.8^{efghijk}$	8.2	75.2^{klm}	10.6
G9	386.7^{lmnop}	1.9	$26.5^{abcdefg}$	2.2	550.1	2671.5	0.2	$0.8^{defghij}$	5.2	$56.1^{ijklmno}$	7.8	27.4^{ijklmn}	12.2	64.9^{ghijk}	10.0	$33.9^{fghijkl}$	16.3	68.2^{ghijkl}	6.8
G10	367.7^{klmno}	2.3	$26.7^{abcdefg}$	2.2	566.9	2416.1	0.2	$0.8^{cdefghij}$	4.2	55.8^{nopq}	5.8	$27.1^{hijklmn}$	6.6	66.0^{hijkl}	7.2	$34.7^{ghijklm}$	8.8	68.1^{ghijkl}	7.3
G11	404.7^{opqrs}	0.4	$28.0^{cdefghi}$	0.0	633.8	3174.3	0.2	0.9^{ijk}	5.0	52.3^{nopq}	11.2	32.5^{op}	9.8	72.2^{klmno}	10.7	40.7^{mno}	9.7	69.9^{ijkl}	14.2
G12	393.0^{mnopq}	3.3	$26.7^{abcdefg}$	4.3	528.5	2476.8	0.2	0.8^{cdefgh}	4.1	$52.7^{efghijkl}$	9.7	$26.0^{ghijklmn}$	10.3	63.2^{fghij}	8.9	$33.6^{fghijkl}$	9.9	$64.9^{fghijkl}$	10.0
G13	257.0^{bcde}	2.0	29.3^{fghi}	5.2	610.5	1265.1	0.5	0.7^{abc}	8.4	34.5^{a}	7.3	14.9^{a}	14.4	42.2^{a}	8.6	19.9^{a}	15.4	47.4^{ab}	7.4
G14	264.7^{cde}	1.3	$28.0^{cdefghi}$	0.0	567.6	1241.5	0.5	0.7^{ab}	6.3	$50.6^{defghijk}$	8.1	21.8^{cdefg}	8.8	60.1^{defghi}	7.2	27.9^{bcdef}	10.4	70.6^{ijklm}	11.3
G15	325.3^{abcd}	0.3	26.0^{abcdef}	0.7	485.9	1591.1	0.3	0.7^{ab}	4.4	$52.3^{defghijkl}$	7.3	22.0^{cdefgh}	4.5	63.2^{fghij}	3.5	28.8^{cdefgh}	2.4	75.5^{ijklm}	10.6
G16	241.4^{hij}	4.5	25.9^{abcdef}	3.9	539.8	907.1	0.6	0.9^{ijk}	6.0	46.3^{cdef}	9.6	$25.5^{efghijklm}$	10.2	57.5^{cdefgh}	8.0	$34.5^{ghijklm}$	11.5	51.3^{bcdefg}	12.9
G17	231.0^{abc}	0.4	28.3^{defghi}	4.1	547.9	812.0	0.7	0.8^{bcdef}	6.8	58.2^{klmnop}	9.2	$27.0^{hijklmn}$	8.7	70.6^{jklmno}	8.5	35.7^{hijklm}	11.2	76.3^{ijklm}	13.4
G18	213.7^{a}	0.5	31.3^{ij}	6.6	641.1	735.0	0.9	0.9^{ghijk}	5.9	$56.3^{ijklmno}$	9.2	29.3^{lmno}	9.7	67.8^{ijklmn}	10.2	38.2^{klmn}	13.3	64.8^{ghijkl}	12.1
G19	214.7^{a}	0.5	30.0^{ghij}	0.0	584.5	683.1	0.9	$0.8^{efghijk}$	6.1	$51.7^{defghijkl}$	9.2	$26.2^{ghijklmn}$	13.4	59.7^{defghi}	9.8	$32.2^{efghijk}$	15.2	$61.4^{cdefghi}$	9.3
G20	217.3^{a}	0.3	28.7^{efghi}	4.0	534.6	647.7	0.8	$0.8^{efghijk}$	8.5	59.2^{lmnop}	11.1	30.5^{nop}	10.0	70.5^{jklmno}	10.8	39.2^{lmno}	12.5	71.0^{ijklm}	17.1
G21	226.0^{abc}	0.8	28.7^{efghi}	4.0	554.0	770.8	0.7	0.8^{cdefg}	4.1	48.5^{cdefgh}	11.2	$23.3^{defghij}$	10.7	59.4^{defghi}	10.7	$31.1^{defghij}$	11.3	$60.7^{cdefghi}$	12.5
G22	216.7^{a}	0.3	31.0^{hij}	3.2	611.9	733.6	0.8	$0.8^{defghij}$	5.6	66.5^{q}	13.4	32.3^{op}	11.0	78.1^{o}	11.5	40.9^{mno}	11.2	81.4^{mn}	16.6
G23	224.7^{ab}	0.5	29.7^{fghij}	5.2	605.9	841.4	0.7	0.8^{bcdef}	9.6	47.9^{cdefg}	13.3	22.3^{cdefgh}	18.2	58.8^{defgh}	10.2	29.9^{defgh}	16.2	61.8^{defghi}	14.1
G24	273.0^{def}	2.6	30.0^{ghij}	3.3	640.9	1473.2	0.4	1.1^{lm}	6.1	63.8^{opq}	7.3	42.4^{s}	10.6	75.9^{mno}	6.2	54.9^{q}	10.9	56.6^{bcdefg}	9.7
G25	441.0^{s}	4.4	33.3^{j}	1.7	724.1	3772.5	0.2	1.1^{lm}	6.0	58.1^{klmnop}	12.1	37.4^{qr}	14.7	66.6^{hijklm}	12.1	45.8^{op}	14.5	52.9^{abcdef}	12.2
G26	322.0^{ghij}	1.4	28.3^{defghi}	2.0	606.3	1958.7	0.3	1.2^{m}	9.9	$49.0^{cdefghi}$	9.9	33.8^{pq}	7.7	60.1^{defghi}	5.7	45.7^{op}	12.0	41.7^{a}	19.1
G27	285.7^{efg}	3.5	$26.3^{abcdefg}$	5.8	502.0	1288.7	0.4	0.7^{a}	3.7	61.6^{mnop}	6.5	$24.8^{efghijkl}$	8.3	75.7^{lmno}	8.2	$33.2^{efghijkl}$	7.9	92.7^{n}	7.7
G28	426.7^{qrs}	1.4	26.0^{abcdef}	0.0	530.5	2677.3	0.2	0.9^{ghijk}	6.4	$52.8^{defghijkl}$	5.0	28.0^{ijklmn}	9.5	60.1^{defghi}	6.5	$34.0^{efghijkl}$	13.5	$58.8^{bcdefghi}$	8.7
G29	358.7^{jklm}	0.7	25.0^{abcde}	4.0	488.9	1986.1	0.2	0.9^{jk}	6.3	46.1^{cdef}	7.2	$25.0^{efghijkl}$	10.8	54.7^{cdef}	10.9	$31.9^{efghijkl}$	16.0	50.6^{abcd}	8.1
G30	433.3^{rs}	0.7	26.0^{abcdef}	0.0	552.5	2985.7	0.2	0.9^{hijk}	5.3	$55.5^{ghijklmn}$	10.0	28.6^{klmno}	13.0	63.2^{fghij}	10.4	$34.8^{ghijklm}$	14.4	$62.3^{defghij}$	9.1

G31	435.7^{rs}	3.7	24.3^{abc}	8.6	450.3	2358.8	0.2	0.8^{efghijk}	5.4	46.1^{cdef}	16.4	25.0^{efghijkl}	15.0	54.7^{cdef}	15.2	31.9^{defghi}	13.5	50.6^{abcd}	19.6
G32	370.7^{klmno}	2.1	25.3^{abcde}	2.3	498.7	2122.8	0.2	0.9^{k}	6.4	49.2^{cdefghi}	15.7	27.4^{ijklmn}	16.8	59.1^{defghi}	13.9	35.7^{hijklm}	16.4	53.4^{abcdef}	17.1
G33	374.0^{klmno}	4.4	25.3^{abcde}	4.6	486.4	2208.7	0.2	0.8^{efghijk}	7.3	49.8^{defghij}	10.6	25.1^{efghijkl}	9.7	59.0^{defgh}	10.5	32.0^{efghijk}	13.6	59.5^{cdefghi}	15.7
G34	361.0^{jklm}	4.7	25.0^{abcde}	4.0	434.1	1726.4	0.3	0.9^{ghijk}	9.3	44.4^{bcd}	11.4	22.8^{cdefghi}	10.4	51.9^{bcd}	10.3	28.9^{cdefgh}	15.6	50.8^{abcde}	18.5
G35	308.5^{fghij}	4.6	26.0^{abcdef}	5.4	479.1	1440.3	0.3	0.8^{cdefghi}	5.0	50.7^{defghijk}	6.2	24.6^{efghijk}	6.3	59.0^{defgh}	7.1	30.7^{defghij}	7.5	62.4^{defghijk}	9.0
G36	401.7^{nopqr}	4.9	24.0^{ab}	4.2	393.0	1910.0	0.2	0.9^{fghijk}	4.6	54.1^{fghijklm}	7.3	27.0^{hijklmn}	12.2	63.8^{fghij}	8.6	34.3^{fghijkl}	13.4	63.6^{efghijkl}	5.8
G37	336.7^{ijk}	4.7	23.0^{a}	4.4	380.1	1398.1	0.3	0.7^{abcd}	6.3	44.5^{bcd}	6.8	19.5^{abcd}	11.8	52.1^{bcde}	6.5	24.6^{abcd}	11.7	60.3^{cdefghi}	7.4
G38	290.3^{efgh}	2.6	24.7^{abcd}	4.7	463.4	1225.8	0.4	0.7^{abcd}	7.8	37.0^{ab}	6.1	16.2^{ab}	13.4	44.2^{ab}	7.6	20.9^{ab}	14.7	50.2^{abcd}	7.1
G39	430.7^{qrs}	4.3	24.0^{ab}	12.5	410.4	2153.5	0.2	0.8^{bcdefg}	11.4	44.8^{bcde}	7.4	20.8^{bcdef}	14.3	52.8^{bcde}	10.3	26.5^{abcde}	16.8	57.1^{bcdefgh}	12.2
G40	436.0^{rs}	2.7	26.3^{abcdefg}	2.2	546.5	2989.4	0.2	0.8^{abcde}	7.9	42.1^{abc}	7.7	18.6^{abc}	12.7	48.7^{abc}	9.9	23.0^{abc}	15.8	55.9^{bcdefg}	9.2
G41	486.7^{t}	2.8	25.3^{abcde}	2.3	505.9	3080.6	0.2	0.7^{abc}	11.6	51.0^{defghijk}	7.5	22.0^{cdefgh}	19.1	59.3^{defghi}	10.6	28.8^{cdefgh}	19.5	69.9^{ijkl}	7.2
G42	374.0^{klmno}	2.7	27.0^{bcdefg}	2.2	545.5	2402.5	0.2	0.8^{cdefghij}	4.9	56.2^{ijklmno}	5.3	27.1^{hijklmn}	8.7	64.9^{ghijk}	6.9	33.6^{fghijkl}	12.3	68.5^{ghijkl}	5.3
G43	352.7^{jkl}	3.8	26.0^{abcdef}	3.9	499.9	1908.9	0.3	0.9^{ijk}	6.0	49.2^{cdefghi}	15.1	26.6^{hijklmn}	16.1	59.7^{defghi}	14.6	35.0^{hijklm}	16.9	54.8^{bcdef}	15.1
G44	387.3^{lmnop}	5.8	27.0^{bcdefg}	0.0	531.9	2805.5	0.2	0.9^{ghijk}	4.3	53.3^{fghijklm}	9.1	28.6^{klmno}	9.6	64.7^{ghijk}	9.5	37.6^{jklmn}	11.0	61.3^{cdefghi}	11.0
G45	355.3^{jkl}	6.7	25.0^{abcde}	0.0	537.9	2391.2	0.2	0.7^{abcd}	6.3	51.1^{defghijk}	6.2	23.0^{cdefghij}	7.7	60.7^{defghi}	8.4	29.5^{cdefgh}	12.0	69.5^{hijkl}	9.9
G46	308.3^{fghi}	1.3	24.0^{ab}	8.3	442.9	1292.4	0.3	0.7^{a}	4.8	49.5^{cdefghi}	10.8	20.8^{bcde}	10.1	61.6^{efghij}	11.2	28.0^{bcdefg}	10.5	75.0^{jklm}	13.9

[1] Mean value of n = 3; [2] Mean value of n = 12; RSD: relative standard deviation; Mean values associated with different small superscript letters indicate significant differences between vital gluten samples within one experimental setup (one-way ANOVA, Tukey test, $p < 0.05$)

Table S3. Scoring system for vital gluten (VG) samples G1-G46 based on the significant parameters of the GlutoPeak and the microscale extension test (PMT, BEM, PMT•BEM, peak180, peak30/peak180, xc1, w, xc3, E_{max}, E_{max}·A_{max}, A_{Rmax}, E_{max} and A_{max}). Points were attributed based on the P25 and P75 interval of Table S1 ("good" = 20 points, "medium" = 10 points and "poor" = 0 points) and weighted by the respective Spearman's correlation coefficients r_S (Table 2).

VG	PMT	BEM	PMT•BEM	peak180	peak30/180	xc1	w	xc3	E_{Rmax}	E_{Rmax}•R_{max}	A_{Rmax}	E_{max}	A_{max}	Total	Identification
G1	5.3	11.2	4.3	4.1	5.1	5.4	6.4	5.1	4.8	7.9	8.8	5.6	9.9	84.1	c
G2	5.3	5.6	4.3	4.1	5.1	5.4	6.4	5.1	9.6	7.9	8.8	11.3	9.9	89.0	c
G3	5.3	5.6	4.3	4.1	5.1	5.4	3.2	5.1	9.6	7.9	8.8	11.3	9.9	85.8	f
G4	0.0	5.6	0.0	0.0	0.0	0.0	0.0	5.1	4.8	4.0	4.4	5.6	5.0	34.5	f
G5	0.0	11.2	0.0	0.0	5.1	5.4	3.2	0.0	9.6	7.9	8.8	11.3	9.9	72.5	c
G6	5.3	11.2	4.3	4.1	5.1	5.4	3.2	0.0	9.6	4.0	4.4	11.3	5.0	72.9	c
G7	5.3	5.6	0.0	0.0	5.1	5.4	6.4	5.1	4.8	4.0	4.4	5.6	5.0	56.7	c
G8	5.3	5.6	4.3	0.0	5.1	5.4	3.2	5.1	9.6	4.0	4.4	11.3	5.0	68.3	f
G9	5.3	5.6	4.3	0.0	5.1	5.4	6.4	0.0	4.8	4.0	4.4	5.6	5.0	55.9	f
G10	5.3	5.6	4.3	4.1	5.1	5.4	3.2	5.1	4.8	4.0	4.4	11.3	5.0	67.6	f
G11	5.3	11.2	0.0	0.0	5.1	5.4	6.4	0.0	9.6	7.9	8.8	11.3	9.9	81.0	c
G12	5.3	5.6	4.3	4.1	5.1	5.4	6.4	5.1	4.8	4.0	4.4	5.6	5.0	65.2	f
G13	10.6	11.2	8.6	8.3	10.2	10.9	6.4	10.2	0.0	0.0	0.0	0.0	0.0	76.3	c
G14	10.6	11.2	8.6	8.3	10.2	10.9	6.4	10.2	4.8	0.0	0.0	5.6	0.0	86.8	c
G15	10.6	5.6	8.6	8.3	10.2	10.9	6.4	10.2	4.8	0.0	0.0	5.6	0.0	81.2	c
G16	10.6	5.6	8.6	8.3	10.2	10.9	3.2	10.2	0.0	4.0	4.4	5.6	5.0	86.5	c
G17	10.6	11.2	8.6	8.3	10.2	10.9	6.4	10.2	9.6	4.0	4.4	11.3	9.9	115.6	c
G18	10.6	11.2	8.6	8.3	10.2	10.9	6.4	10.2	4.8	7.9	8.8	11.3	9.9	119.1	c
G19	10.6	11.2	8.6	8.3	10.2	10.9	3.2	10.2	4.8	4.0	4.4	5.6	5.0	96.9	c
G20	10.6	11.2	8.6	8.3	10.2	10.9	6.4	10.2	9.6	7.9	8.8	11.3	9.9	123.9	c
G21	10.6	11.2	8.6	8.3	10.2	10.9	6.4	10.2	4.8	0.0	0.0	5.6	5.0	91.7	c
G22	10.6	11.2	8.6	8.3	10.2	10.9	6.4	5.1	9.6	7.9	8.8	11.3	9.9	118.9	c
G23	10.6	11.2	8.6	8.3	10.2	10.9	6.4	10.2	4.8	0.0	0.0	5.6	5.0	91.7	c
G24	10.6	11.2	8.6	8.3	10.2	10.9	0.0	10.2	9.6	7.9	8.8	11.3	9.9	117.5	c
G25	0.0	11.2	0.0	0.0	5.1	5.4	3.2	5.1	9.6	7.9	8.8	11.3	9.9	77.6	c
G26	10.6	11.2	4.3	4.1	10.2	10.9	6.4	10.2	4.8	7.9	8.8	5.6	9.9	105.0	c
G27	10.6	5.6	8.6	8.3	10.2	10.9	6.4	10.2	9.6	4.0	4.4	11.3	5.0	105.0	c
G28	0.0	5.6	0.0	0.0	5.1	5.4	3.2	5.1	4.8	4.0	8.8	5.6	5.0	52.6	f
G29	10.6	5.6	8.6	4.1	5.1	10.9	6.4	0.0	0.0	4.0	4.4	0.0	5.0	64.7	f
G30	0.0	5.6	0.0	0.0	0.0	5.4	6.4	0.0	4.8	7.9	8.8	5.6	5.0	49.6	c
G31	0.0	0.0	4.3	4.1	5.1	5.4	3.2	5.1	0.0	4.0	4.4	0.0	5.0	40.6	f
G32	5.3	5.6	4.3	4.1	5.1	5.4	6.4	10.2	4.8	4.0	4.4	5.6	9.9	75.2	c
G33	5.3	5.6	4.3	4.1	5.1	0.0	0.0	5.1	4.8	4.0	4.4	5.6	5.0	53.3	c

G34	5.3	5.6	4.3	4.1	10.2	0.0	0.0	5.1	0.0	0.0	0.0	0.0	0.0	34.7	f
G35	10.6	5.6	8.6	8.3	10.2	10.9	3.2	10.2	4.8	4.0	4.4	5.6	5.0	91.3	f
G36	5.3	0.0	4.3	4.1	5.1	0.0	3.2	5.1	4.8	4.0	4.4	5.6	5.0	50.9	c
G37	10.6	0.0	8.6	8.3	10.2	10.9	3.2	5.1	0.0	0.0	0.0	0.0	0.0	56.9	c
G38	10.6	0.0	8.6	8.3	10.2	10.9	6.4	5.1	0.0	0.0	0.0	0.0	0.0	60.1	f
G39	0.0	0.0	4.3	4.1	5.1	5.4	3.2	5.1	0.0	0.0	0.0	0.0	0.0	27.3	c
G40	0.0	5.6	0.0	0.0	0.0	5.4	3.2	5.1	0.0	0.0	0.0	0.0	0.0	19.3	c
G41	0.0	5.6	0.0	0.0	0.0	5.4	3.2	0.0	4.8	0.0	0.0	5.6	0.0	24.7	c
G42	5.3	5.6	4.3	4.1	5.1	5.4	3.2	0.0	4.8	4.0	4.4	5.6	5.0	56.9	c
G43	10.6	5.6	4.3	4.1	10.2	10.9	3.2	5.1	4.8	4.0	4.4	5.6	5.0	77.8	f
G44	5.3	5.6	4.3	0.0	0.0	5.4	3.2	5.1	4.8	4.0	8.8	5.6	9.9	62.1	f
G45	10.6	5.6	8.6	4.1	5.1	5.4	3.2	5.1	4.8	0.0	0.0	5.6	0.0	58.2	f
G46	10.6	0.0	8.6	8.3	10.2	10.9	6.4	10.2	4.8	0.0	0.0	5.6	0.0	75.6	f

c: correct; f: false
